# Capillary leak-syndrome triggered by Maripa virus in French Guiana: case report and implication for pathogenesis

**DOI:** 10.1186/s12879-019-3887-5

**Published:** 2019-03-15

**Authors:** Hatem Kallel, Séverine Matheus, Claire Mayence, Stéphanie Houcke, Cyrille Mathien, Anne Lavergne, Didier Hommel

**Affiliations:** 1 Service de Réanimation Polyvalente, Centre Hospitalier Andrée Rosemon de Cayenne, Avenue des Flamboyants, 6006 97306 Cayenne, BP French Guiana; 2 Centre National de Référence des Hantavirus, Laboratoire associé, Institut Pasteur de la Guyane, Cayenne, French Guiana; 3 Laboratoire des Interactions Virus-Hôtes, Institut Pasteur de la Guyane, Cayenne, French Guiana

**Keywords:** Hantavirus, Maripa, Pulmonary syndrome, Capillary leak-syndrome

## Abstract

**Background:**

We report hereby a severe case of Hantavirus Pulmonary Syndrome” (HPS) induced by Maripa virus in French Guiana and describe the mechanism of severity of the human disease.

**Case presentation:**

A 47-year- old patient started presenting a prodromic period with fever, dyspnea, cough and head ache. This clinical presentation was followed by a rapid respiratory, hemodynamic and renal failure leading to admission in the ICU. Biological exams revealed an increased haematocrit level with a paradoxical low protein level. Echocardiographic and hemodynamic monitoring showed a normal left ventricular function with low filling pressures, an elevated extravascular lung water index and pulmonary vascular permeability index. These findings were compatible with a capillary leak-syndrome (CLS).

**Conclusions:**

The severity of HPS caused by the virus Maripa in French Guiana can be explained by the tropism of hantavirus for the microvascular endothelial cell leading to a CLS.

## Background

Hantavirus generally stands as a rodent-borne virus infection. Rodent shed the virus in their droppings, saliva and urine. Human infection occurs after breathing air contaminated by the virus. This can result on a severe respiratory syndrome named ‘Hantavirus pulmonary syndrome’ (HPS) which can be associated to cardiac failure leading to ‘Hanta virus cardio-pulmonary syndrome’ (HCPS) [1].

In French Guiana, 5 cases of HPS due to a hantavirus named Maripa virus were diagnosed between 2008 and 2017 [2, 3]. We report hereby the 6th human case of HPS diagnosed in French Guiana and describe the mechanism of severity of the human disease.

## Case presentation

Our 47-year-old patient with a history of tobacco, alcohol, and illicit-drug consumption was admitted to the ICU for fever (39 °C), tachycardia (152 beat/min), hemodynamic shock (Blood arterial Pressure was 78/63 mmHg), acute respiratory distress syndrome (ARDS; PaO_2_/FiO_2_ ratio was 62) with signs of intra-alveolar haemorrhage and, acute renal failure. Symptoms including headache, fever, cough and dyspnea leading to respiratory failure started 6 days before admission. The treatment consisted of crystalloids and norepinephrine infusion as well as mechanical ventilation support and continuous renal replacement therapy (RRT).

Initial laboratory testing showed renal impairment (urea nitrogen at 9.2 mmol/L, serum creatinine at 168 μmol/L), a rise in inflammatory parameters (Leucocytes count at 17.8 G/L and C-Reactive Protein at 145 mg/L), an increased haematocrit level (46.9%), thrombocytopenia (107 G/L), a low protein level (49 g/L) and cellular dysoxia (Lactates dosage at 3.6 mmol/L). All other biological tests including the dosage of hepatic, muscular and cardiac enzymes were normal on admission. Chest X-Ray showed bilateral alveolar infiltrates and bilateral pleural effusion. Transthoracic echocardiography showed normal and homogeneous left ventricular contractility with low filling pressures, with an aortic Velocity Time Integral (VTI) of 10 cm (normal: 18–25 cm), a normal right heart function with Tricuspid annular plane systolic excursion (TAPSE) at 14 mm (normal: 15–20 mm), and a small pericardial effusion. Hemodynamic monitoring using transpulmonary thermodilution (PiCCO system; Pulsion Medical Systems SE, Feldkirchen, Germany) showed a cardiac output (CO) of 4.2 l/min (normal: 4–8 l/min), a global end diastolic volume index (GEDVI) of 610 ml/m^2^ (normal: 680- 800 ml/m^2^), an extravascular lung water index (EVLWI) of 26.7 ml/kg (normal: 3–7 ml/kg), and a pulmonary vascular permeability index (PVPI) of 6.3 (normal: 1.0–3.0). Chest computed tomography showed vessels enlargement, peribronchial cuffing, bilateral Kerley lines, alveolar edema, and abundant right pleural effusion needing pleural drainage. The protein level in the pleural fluid was 43 g/l and the microbiological culture was sterile. The viral investigations (IgM and RT-PCR in the blood) confirmed an acute infection by Hantavirus. The complete RNA coding sequence of the S RNA segment (GenBank accession no. MG785209) was also generated and compared with those of the other 5 previous hantavirus cases from French Guiana using a Bayesian approach. This S RNA sequences showed a nucleotide identity of 95.9 to 99.5% with the five other previously described sequences of the Maripa virus belonging to the Rio Mamoré clade. Phylogenetic relationships were inferred from alignment with 1308 nt of the S segment [4]. Laboratory testing concerning other infectious agents (bacteria, fungal or parasite) were negative.

**Fig. 1:**
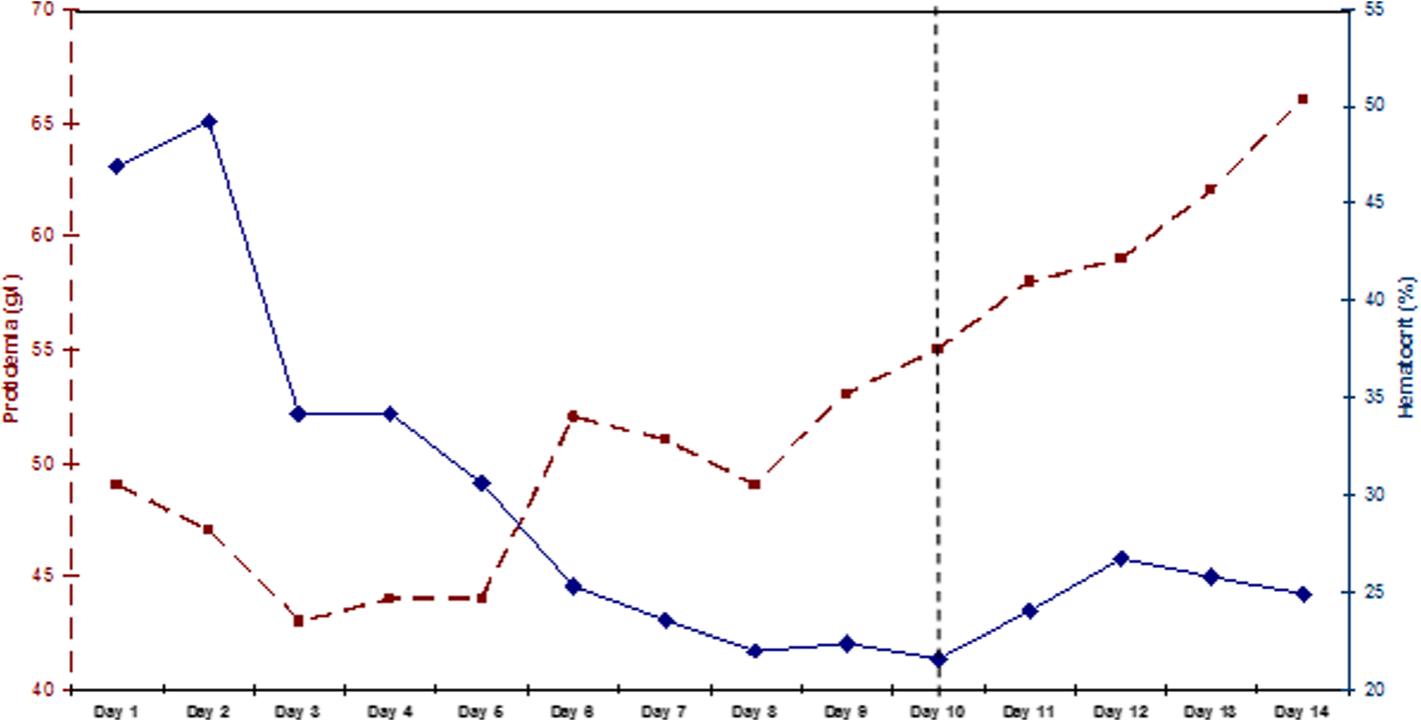
Evolution of the serum protein and hematocrit levels during the first 14 days (norepinephrine was stopped at day 10)

Overall, the patient’s management included mechanical ventilation, norepinephrine, fluid infusion, sedation (midazolam and sufentanil), curarisation (cisatracrium), broad spectrum antibiotics, corticosteroids, and renal replacement therapy. Concerning the outcome, the patient recovered gradually with a concomitant rise in serum protein level and a decrease in haematocrit concentration (Fig. 1). He was weaned from mechanical ventilation at day 18, norepinephrine was stopped at day 10 and RRT at day 21. He fully recovered and left hospital on the 23th day. The patient was examined in the outpatient clinic three weeks after discharge and his clinical examination was normal.

## Discussion and conclusions

We report here a human case of acute Maripa virus related pulmonary syndrome managed in the ICU of French Guiana with a clear evidence of associated capillary leak syndrome responsible for the severity of the disease.

In case of hantavirus infection, initial symptoms include fever, myalgia and headache followed by gastrointestinal symptoms such as abdominal pain, vomiting and diarrhea [1]. This stage lasts around 5 to 7 days before the onset of respiratory failure, hypotension and cardiovascular shock. Thrombocytopenia with haemorrhagic symptoms and renal injury are also frequently reported [3].

In our case, symptoms recorded at admission were compatible with acute infection by hantavirus and required admission to the ICU. The mechanism which may explain the severity of the disease is the tropism of hantavirus for the microvascular endothelial cell [5]. This tropism causes microvascular hyperpermeability with fluid and proteins leakage leading to hypovolemia and to a non cardiogenic pulmonary oedema. Biologically, we observe an increased haematocrit level due to hemoconcentration, and a paradoxical reduced serum proteins level secondary to the transfer of proteins from the vessel to the interstitial space.

Under normal conditions, the endothelium plays the role of a selective permeable barrier to regulate plasma fluid exchange, as well as molecules and cells trafficking. Disruption of cell junctions, with combination of cell retraction process, lead to the loss of the vascular endothelium barrier function. In such conditions, fluids and proteins infusion are ineffective because of the immediate leakage to the interstitial space with a worsening of the respiratory failure without any efficacy on the hemodynamic state. This mechanism is similar to that reported by Clarkson in 1960 and is explained by a plasma leakage [6] which was also described in arbovirus infections where the diagnosis was based on thoraco-abdominal sonography and scanography [7–9].

In our patient, hemodynamic investigations using echocardiography and the PICCO system showed hypovolemia with low filling pressures and without any ventricular dysfunction. An elevated amount of extravascular lung water as well as an increased vascular permeability were also observed. This result is confirmed by the chest CT scan findings, showing a large amount of water in the alveoli, in the perivascular and in the pleural space. The pleural effusion was exudative and contained a high quantity of protein which can be explained by a protein leakage rather than by an inflammatory origin.

The pathogenesis of capillary leakage remains undefined. Some evidence suggest that hantavirus disease pathogenesis is immunologically mediated by cytotoxic T lymphocytes and other immune cells in target organs producing inflammatory cytokines. Overall, three hypotheses have been reported to explain the mechanism of increased capillary permeability involved in hantavirus infection: a) the attack of infected endothelial cells by virus-specific cytotoxic T lymphocytes (CTLs), b) TNF-α production by infected monocyte/macrophages and finally c) the direct effect of the virus on the endothelial cell functions [5, 10]. Bradykinin, a potent inflammatory and vasoactive nonapeptide generated by kallikrein at the sites of tissue injury is supposed to be the key mediator of the vascular leakage resulting from hantavirus infection. It acts by disrupting inter-endothelial junctions and causes changes in vascular tone. Two patients with severe capillary leak syndrome caused by a Puumala hantavirus infection were successfully treated with a bradykinin receptor antagonist [11, 12]. Experimental data demonstrating the plasma kallikrein-kinin system activation during hantavirus infection were also reported [13]. In the same way, the intensity of the inflammatory syndrome was correlated to the importance of the capillary leakage and to the level of thrombocytopenia [14].

Despite abundant literature on hantavirus, few reports have focused on the aetiology of shock in severe hantavirus infected patients. Many studies assume that the shock associated to hantavirus pulmonary syndrome is cardiogenic and hantavirus induces a typical myocarditis. These data were based on the examination of postmortem tissue from human HPS cases [15]. However, in our case, myocardial dysfunction was neither observed during echocardiography and nor during PICCO investigation. Any inotropic agent support has been needed. In addition, Troponine levels were normal despite severe shock. Consequently, we think that Maripa virus is more responsible for HPS rather than HCPS. Such a finding is important as it raises the question about the effectiveness of extracorporal membrane oxygenation (ECMO) in patients presenting Maripa Virus infection with severe shock.

We conclude that HPS secondary to Maripa virus infection in French Guiana can cause severe damages leading to Multi Organ Failure. The severity of the disease may be explained by a dysregulated inflammatory and immune reaction causing a severe capillary leakage without cardiac involvement. Physicians should be aware of HPS occurring in French Guiana and any immediate management in the ICU should be considered.

## Abbreviations

ARDS: Acute Respiratory Distress Syndrome
CO: Cardiac Output
ECMO: Extra Corporal Membrane Oxygenation
EVLWI: Extravascular Lung Water Index
GEDVI: Global End Diastolic Volume Index
HCPS: Hantavirus Cardio-Pulmonary Syndrome
HPS: Hantavirus Pulmonary Syndrome
PICCO: Pulse Contour Cardiac Output
PVPI: Pulmonary Vascular Permeability Index
RRT: Renal Replacement Therapy
RT-PCR: Reverse Transcription Polymerase Chain Reaction
TAPSE: Tricuspid Annular Plane Systolic Excursion
VTI: Velocity Time Integral
